# EROS 2.0 study: evaluation of two interventional radiotherapy (brachytherapy) schedules for endometrial cancer: a comparison of late vaginal toxicity rates

**DOI:** 10.1007/s11547-022-01455-y

**Published:** 2022-01-29

**Authors:** Valentina Lancellotta, Gabriella Macchia, Nicola Dinapoli, Rosa Autorino, Maura Campitelli, Alessia Nardangeli, Alessandra Salvati, Bruno Fionda, Calogero Casà, Patrizia Cornacchione, Angeles Rovirosa, György Kovács, Alessio Giuseppe Morganti, Maria Gabriella Ferrandina, Maria Antonietta Gambacorta, Luca Tagliaferri

**Affiliations:** 1 UOC Radioterapia Oncologica, Dipartimento di Diagnostica per Immagini, Radioterapia Oncologica ed Ematologia, Fondazione Policlinico Universitario “A. Gemelli” IRCCS, 00128 Rome, Italy; 2 Gemelli Molise Hospital, Radiation Oncology Unit, Università Cattolica del Sacro Cuore, 86100 Campobasso, Italy; 3 Department of Radiation Oncology, Hospital Clinic i Universitari, Fonaments Clinics Department, University of Barcelona, 08036 Barcelona, Spain; 4 Università Cattolica del Sacro Cuore, 00128 Roma, Italy; 5 Radiotherapy Center, IRCCS Azienda Ospedaliera Universitaria di Bologna, 40138 Bologna, Italy; 6 DIMES, Alma Mater Studiorum University of Bologna, 40138 Bologna, Italy; 7 Woman, Child and Public Health Department, Fondazione Policlinico Universitario “A. Gemelli”, IRCCS, Roma, Italy; 8 Istituto di Radiologia, Università Cattolica del Sacro Cuore, 00128 Rome, Italy

**Keywords:** Endometrial cancer, Fractionation, Vaginal late toxicity, Brachytherapy, Interventional radiotherapy

## Abstract

**Background:**

To compare the late toxicity rates after two different high dose rate (HDR) adjuvant intravaginal interventional radiotherapy (IRT-brachytherapy) dose schedules in stage I-II endometrial cancer.

**Methods:**

Stage I-II patients with endometrial cancer treated with surgery (with or without lymphadenectomy) and adjuvant HDR-IRT between 2014 and 2020 were included in this analysis. Patients were treated with two schedules. In the first cohort (C1), 21 Gy were delivered in three weekly fractions (7 Gy) prescribed 0.5 cm from the applicator surface. In the second cohort (C2), 24 Gy were delivered in four weekly fractions (6 Gy). The clinical target volume was the upper third of the vagina for C1 and the upper 3 cm for C2. HDR-IRT technique and point prescription (5 mm depth from the applicator surface) were the same for all patients. Vaginal toxicity was scored according to the CTCAE 5.0 scale in terms of the presence *versus* absence of any toxicity grade. The correlation among toxicity and clinical covariates (age, lymphadenectomy, fractionation, stage) was tested by Pearson correlation test (univariate) and by logistic regression (multivariable).

**Results:**

114 stage I and three stage II patients, median age 62 (range: 32–85) years, were included in this analysis. The mean follow-up was 56.3 months in C1 (40–76) and 20 months in C2 (8–42). Vaginal late toxicity was recorded in 40 and 15 patients in C1 and 2, respectively. Age, lymphadenectomy, and fractionation were significantly correlated with toxicity at univariate analysis (p value = 0.029, 0.006, and 0.002, respectively), while stepwise logistic regression confirmed only age and fractionation as significantly correlated parameters (p value = 0.02 and 0.001, respectively). Three-year local relapse-free, distant metastasis-free and cause-specific survival rates were 96.6%, 94.8%, and 99.1%, respectively.

**Conclusions:**

This analysis showed lower vaginal late toxicity rate in C2 compared to C1.

## Introduction

Endometrial cancer (EC) is the most frequent gynecological cancer and the fourth most common tumor in women [1]. About 70–80% ECs are confined to the uterus, and 80% are endometrioid adenocarcinomas. Total abdominal hysterectomy and bilateral salpingo-oophorectomy, with or without pelvic and paraaortic lymph node dissection, represent the upfront treatment [2]. According to patient- and tumor-related risk factors, adjuvant pelvic external beam radiotherapy (EBRT) or intravaginal interventional radiotherapy (IRT, i.e., brachytherapy) is recommended in patients with intermediate-high risk of relapse [1, 2].

Starting from the assumption that the vagina is the most frequent (nearly 75%) site of recurrence in non-irradiated patients [3–5], the Postoperative Radiotherapy in Endometrial Cancer trial (PORTEC 2) showed that IRT is as effective as pelvic EBRT in preventing vaginal recurrences, with fewer adverse effects and improved quality of life [6]. More than 24 adjuvant vaginal cuff IRT regimens, with different dose/fractionation schedules, were effective in minimizing vaginal recurrences rates to 2% or less [7, 8]. The most commonly used schedules are 30 Gy in five fractions (MD Anderson Cancer Center), 24 Gy in six fractions (Brigham and Women's Hospital/Dana-Farber Cancer Institute) [9–11], and 21 Gy in three fractions at 0.5 cm depth, or 30 Gy in five fractions to the applicator surface (American Brachytherapy Society) [8].

Even though all reported IRT schedules lead to excellent oncological outcomes, the fractionation schedule bearing the lowest vaginal toxicity rate remains undefined.

To clarify this issue, herein, we report the late vaginal toxicity rates in EC patients treated with two different adjuvant high dose rate (HDR) IRT schedules.

## Material and methods

### Endpoints

The primary endpoint of the study was late vaginal toxicity, defined as any toxicity occurring six months after completion of HDR-IRT. Secondary endpoints included the comparison in terms of loco-regional recurrence (defined as any vaginal or pelvic recurrence), distant recurrence (defined as any distant failure), and cancer-specific survival (i.e., the time from treatment to cancer-related death).

### Inclusion criteria

Data were retrieved from Spider's Net [12], the hospital intranet multidimensional electronic database. FIGO 2009 Stage I-II EC patients treated with total abdominal hysterectomy and bilateral salpingo-oophorectomy (with/without pelvic/paraaortic lymph node dissection) followed by adjuvant vaginal HDR-IRT were included. Patients with non-endometrial histotype EC were excluded. Informed consent was obtained from all subjects involved in the study. Furthermore, we considered an observation period after IRT (follow-up > 6 months) as mandatory for the inclusion in this analysis.

### Interventional radiotherapy

Vaginal applicator diameter ranged from 2.0 to 3.5 cm (median 3 cm). OncentraBrachy treatment planning system and MicroSelectron (Elekta, Stockholm, Sweden) device with a 192-Ir source were used to plan and treat the first cohort of patients (cohort 1–C1), respectively. The proximal third of the vagina was irradiated with a HDR-IRT schedule based on 21 Gy in three weekly fractions (7 Gy) prescribed 0.5 cm from the applicator surface. OncentraBrachy treatment planning system and a Flexitron (Elekta, Stockholm, Sweden) device with a 192-Ir source were used to treat the second cohort of patients (cohort 2 – C2). The upper 3 cm of the vagina was irradiated by a HDR-IRT schedule of 6 Gy per fraction/weekly (total dose 24 Gy) prescribed 0.5 cm from the applicator surface.

### Follow-up

Follow-up included complete clinical and pelvic examinations every four months for the first two years, every six months for the following three years, and then once a year. The CTCAE v. 5 scale was used a posteriori to score the vaginal toxicity, as summarized in Table 1.

**Table 1:** Common Terminology Criteria for Adverse Events (CTCAE) Version 5.0

CTCAE Term	Grade 1	Grade 2	Grade 3	Grade 4	Grade 5
Vaginal dryness	Mild vaginal dryness not interfering with sexual function	Moderate vaginal dryness interfering with sexual function or causing frequent discomfort	Severe vaginal dryness resulting in dyspareunia or severe discomfort	–	–
Vaginal stricture		Vaginal narrowing and/or shortening not interfering with physical examination	Vaginal narrowing and/or shortening interfering with the use of tampons, sexual activity or physical examination	–	–
Telangiectasia	Telangiectasias covering < 10% BSA	Telangiectasias covering ≥ 10% BSA; associated with psychosocial impact	–	–	–

Vaginal cytology and abdominopelvic ultrasound were carried out every six months over the first two years and then yearly. Contrast-enhanced computed tomography or magnetic resonance imaging scans were performed at clinician’s request.

### Statistical analysis

Statistical analysis has been performed by R statistical software v. 4.0.3. Homogeneity among groups was tested using the t-test for numerical variables (previously tested for normality by Shapiro test), chi-square for factors, and Fisher test for binary variables. Grade 2 vaginal toxicity was considered as the main outcome due to clinical relevance and to the small number of grade 3 events in our case series (see results). The correlation among > Grade 2 toxicity and clinical variables (patient’s age, pathological tumor stage, lymphadenectomy, and IRT fractionation) was calculated using the Kendall correlation test. Using the variables showing high correlation (see results), after checking the absence of cross-correlation by Kendall test, a logistic regression multivariable model was computed to assess the dependance of toxicity on the related variables. The model performance was tested by area under the receiver operating characteristic curve (AUC) and calibration with Hosmer–Lemeshow test. Finally, a nomogram was edited to easily calculate the probability of toxicity. In all statistical tests, a p value < 0.05 was considered as significant.

## Results

### Patient’s characteristics

One hundred seventeen EC patients underwent vaginal IRT after surgery and were included in the retrospective analysis. A first cohort (21 Gy/3 fractions: C_1_) of 60 patients, median age 63 years (range 32–85), were treated from 2014 to 2017 and had a median follow-up of 56.3 months (range 40–76). A second cohort (24 Gy/4 fractions: C_2_) included 57 patients, median age 62 years (43–86), who were treated from 2018 to 2020 and had a median follow-up of 20 months (range 8–42). The difference in follow-up time between the two groups was statistically significant (p value < 0.001; log-rank). Except for this, the two cohorts were well balanced in terms of stage, grading, and risk factors as detailed in Table 2.

**Table 2:** Clinical and pathologic characteristics of the patients: in brackets percentages. P value column shows results of statistical tests between the two groups. Statistical tests are: (1) T-test, (2) Fisher test, (3) Log-rank test, (4) X^2^test, (5) Mann–Whitney

Variable	Group 1			Group 2			P-Value
Age	60			57			0.750^(1)^
Lymphadenectomy	No 22 (18.8)	Yes 38 (32.5)		No 42 (35.9)	Yes 15 (12.8)		< 0.001^(2)^
Lymph nodes removed (median)	15			3			0.001^(5)^
Toxicity	No 26 (23.9)	Yes 34 (27.4)		No 43 (36.8)	Yes 14 (12.0)		0.002^(2)^
Recurrence	No 57 (48.7)	Yes 3 (2.6)		No 56 (47.9)	Yes 1 (0.8)		0.619^(2)^
Follow-Up (median, months)	56.3			20.0			< 0.001^(3)^
Stage	Ia 19 (16.2)	Ib 39 (33.3)	II 2 (1.7)	Ia 24 (20.5)	Ib 33 (28.5)	II 0 (0.0)	0.222^(4)^

Stage IB and Grade 2 were prevalent in both groups. However, differences between cohorts were found in terms of number of removed lymph nodes (17 [range, 1–54] in C_1_ *versus* 3 [range, 1–34] in C_2;_ Wilcoxon-test: p value = 0.001).

### Toxicity

Late vaginal toxicity was recorded in 32 (53.3%) and 14 (24.6%) patients in C_1_ and C_2_ (Fisher test: p value = 0.002), respectively. No severe late toxicity was observed except for one Grade 3 vaginal stricture in C_1_. Details about vaginal toxicity are shown in Table 3.

**Table 3:** Late vaginal toxicities

Type of late toxicity	21 Gy/3 fractions	24 Gy/4 fractions
vaginal strictures	G1: 12 (20%) G2: 2 (3.3%) G3: 1 (1.6%)	G1: 1 (1.7%) G2: 1 (1.7%) G3: 0 (0%)
Vaginal dryness	G1: 21 (35%) G2: 2 (3.3%)	G1: 9 (15.7%) G2: 2 (3.5%)
Telangiectasia	G1: 2 (3.3%)	G1: 2 (3.5%)

*Gy* gray, *G* grade

**Fig. 1:**
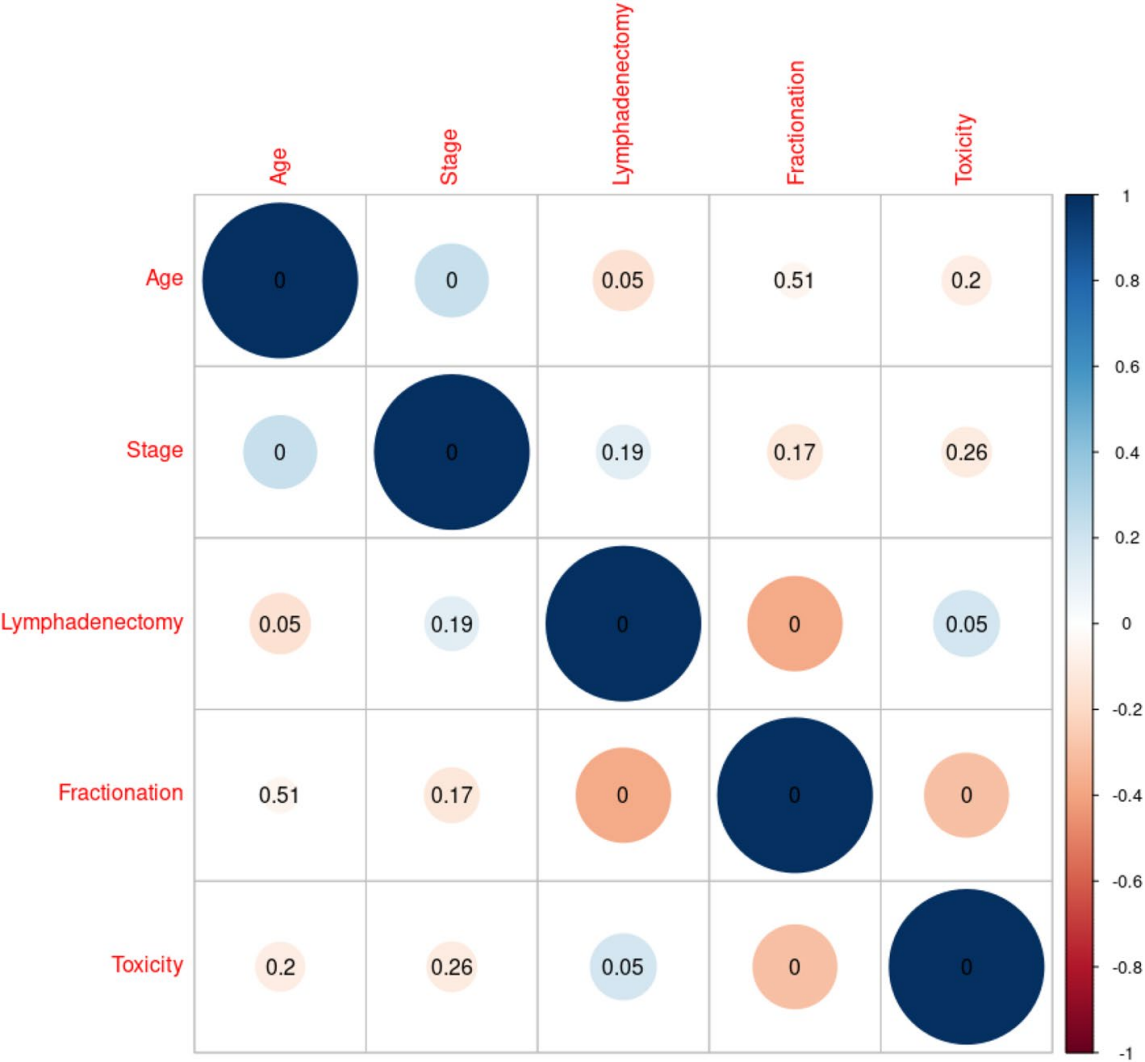
Cross-correlation matrix among variables and toxicity. Blue circles show positive correlation, and red circles show negative correlation. Kendall test p value is shown over the circles in the matrix

Late vaginal toxicity, in C_1_ and C_2_, was recorded after a median interval of 13 months (range 8–35 months) and 12 months (range 6–21 months) from IRT, respectively. The cross-correlation matrix among variables is shown in Fig. 1.

**Fig. 2:**
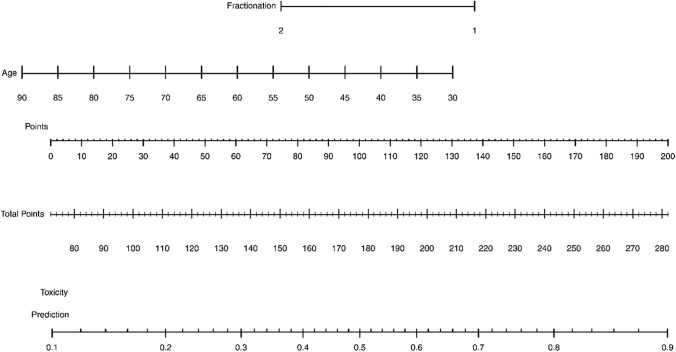
Nomogram for toxicity probability prediction. The two fractionation groups are shown by numbers: 1—C_1_ (21 Gy at 7 Gy/fr), 2 – C_2_ (24 Gy at 6 Gy/fr)

Univariate analysis showed that lymphadenectomy (p value = 0.006) and IRT higher dose/fraction (p value = 0.002) were significantly correlated with higher probability to develop late vaginal toxicity. At stepwise logistic regression, both older age (p value = 0.02) and higher dose/fraction (p value = 0.001) were significantly correlated with vaginal late toxicity (Table 4). Figure 2 shows a nomogram, plotted based on the logistic regression, with the aim to calculate the overall risk of toxicity. The AUC of the logistic regression was 0.705.

**Table 4:** Logistic regression results

Deviance Residuals				
Min	1Q	Median	3Q	Max
− 1.6291	−1.0345	− 0.6297	1.0584	2.0856
Coefficients				

	Estimate	Std. Error	z value	**Pr( >|z|)**
(Intercept)	4.67771	1.54578	3.026	**0.00248**
Age	− 0.04882	0.02102	− 2.323	**0.02019**
Fractionation	− 1.31577	0.41087	− 3.202	**0.00136**

Coefficient of fractionation was used considering this as “numeric” variable, so it is − 1.31577 for C1 and double (− 2.63154) for C2. Bold values are p-values

Null deviance: 159.72 on 116 degrees of freedom

Residual deviance: 143.87 on 114 degrees of freedom

AIC: 149.87

### Outcomes and survival

In C_1,_ three (5%) patients had vaginal cuff relapse, one (1.7%) showed pelvic nodal recurrence, and two (3.3%) had lung or bone metastases. In C_2_, one (1.7%) patient had vaginal cuff relapse, one (1.7%) showed pelvic nodal recurrence, and two (3.5%) developed distant metastases (lung and bone) or peritoneal carcinomatosis. Overall, 3-year local relapse-free survival was 96.6% (95% confidence interval [CI]: 88.1–96.7). At univariate analysis, less than 12 removed lymph nodes were significantly correlated with higher probability of loco-regional recurrence (HR: 7.057; 95% CI: 1.6–29.5; p = 0.008). Moreover, multivariable Cox’s regression analysis confirmed this correlation (HR: 6.952; 95% CI: 1.591–30.385; p = 0.010). To date, 114 (92%) patients are alive and disease-free, while three patients died from EC (one patient; 0.8%) or other causes (two patients; 1.7%). Overall, 3 years distant metastasis-free survival and cause-specific survival were 94.8% (95% CI: 93.2–99.3) and 99.1% (95% CI: 93.5–99.5), respectively. There is no significant difference in local relapse-free survival rates, distant metastasis-free survival and cause-specific survival between the two groups.

## Discussion

Although IRT is an established EC adjuvant treatment, there is a lack of clear consensus on the best dose/fractionation regimen, particularly for vaginal HDR-IRT. Moreover, only few comparative studies analyzed the impact of HDR-IRT dose/fractionation on late vaginal toxicity [13–19]. Due to the lower vaginal toxicity rate compared to 21 Gy in 3 weekly fractions, the results of the present series support the use of a IRT regimen based on 6 Gy per fraction/weekly (total dose 24 Gy) and prescribed 0.5 cm from the applicator surface.

Based on the American Brachytherapy Society [9] recommendations for HDR-IRT, different dose/fractionation regimens produce similar results. It is difficult to compare our results with the literature data due to lack of long-term follow-up outcomes, use of different toxicity scales, and under-reporting of low-grade toxicity resulting in underestimated morbidity in the published series. Regarding late toxicity, the available literature data showed vaginal HDR-IRT as very well tolerated with the main side effects, using different scores morbidity, consisting of Grade 1–2 toxicity though with a wide incidence range (7.5%-27.7%) [20, 21]. Grade 3–4 late vaginal toxicity was reported only in a few cases with less than 2% rate [6].

Due to significant regimens heterogeneities, we tried to compare our results with trials using similar schedules. Rovirosa et al. reported HDR-IRT late toxicity results in 42 patients who received four fractions with a dose/fraction of 5–6 Gy/weekly. Five patients developed G1 (2.3%) and two patients G2 (4.6%) late vaginal toxicity [13]. In a posterior study, from 2003 to 2015, same authors compared three different schedules of postoperative IRT delivered to 146 patients with intermediate-risk EC. Forty-one patients received six 4–6 Gy fractions at 3–4 fractions per week, 59 patients received four 5–6 Gy fractions daily, and 46 patients received three 6 Gy fractions in three consecutive days. Vaginal Grade 1 and Grade 2 late toxicity using the objective criteria of LENT-SOMA ranged from 8.7%-19.5% and 9.8%-19.6%, respectively [14]. Nevertheless, this score of toxicity offers higher values of vaginal complications in comparison to CTCAE scores. Another analysis, Rovirosa et al. in 43 patients with a median follow-up of 51 months, using the same scores of toxicity, showed a 46.5% G1 (mainly telangiectasias) and 4.7% G2 late vaginal toxicity using 3 fractions of 6 Gy or 2 fractions of 7.5 Gy administered daily [14]. Chong et al. delivered 22 Gy in four fractions/twice a week (5.5 Gy per fraction at a depth of 0.5 cm from the applicator surface), reporting vaginal stenosis in 13% of cases [15].

Using a 24 Gy in four weekly fractions schedule, we reported Grade 1 vaginal stricture, vaginal dryness, and telangiectasia in 1.7%, 15.7%, and 3.5% of patients, respectively. Moreover, Grade 2 vaginal stricture, vaginal dryness, and telangiectasia were recorded in 1.7%, 3.5%, and 3.5% of patients, respectively. No late Grade 3–4 toxicity was registered.

The Grade 2 late vaginal toxicity rate reported in our cohorts is lower compared to other series [13–19]. It is well known the rate and severity of late vaginal toxicity are related to the dose prescription point and the treated vaginal length [14, 16, 19, 22, 23]. Moreover, the larger the cylinder size, the smaller the dosimetric differences between different prescriptions points [19]. Therefore, when using 2-cm diameter cylinders, the shift from surface to 0.5 cm depth prescription translates into a dosimetric increase ranging between 120 and 445% of the dose prescription. The relatively low toxicity rate recorded in our series could be related to the choice of the largest applicator diameter according to the patient anatomy to minimize the air gap between the applicator surface and the vaginal mucosa and the consequent lower dose to the vaginal mucosa [19, 22].

The second risk factor for late toxicity is the length of irradiated vagina. Many studies reported that vaginal toxicity is correlated with active length ≥ 5 cm and older age [14, 16, 23, 24]. Historically, in our institution, the clinical target volume was defined as the proximal third of the vagina plus 5 mm isotropic margin. More recently, the clinical target volume was defined as the proximal 3 cm of the vagina. A shorter length may explain the lower toxicity found with the 24 Gy in four fractions schedule. Moreover, in our series, older age (p = 0.02) and higher dose/fraction (p = 0.001) were correlated with the development of late vaginal toxicity.

Overall, the 3-year local relapse-free survival was 96.6% (95% confidence interval [CI]: 88.1–96.7), the 3 years distant metastasis-free was 94.8% (95% CI: 93.2–99.3), and 3 years cause-specific survivals were 99.1% (95% CI: 93.5–99.5). These figures are in line with the results reported by other authors [3–6, 9].

As in other clinical settings, the prescription of adjuvant IRT in EC needs to balance risk factors, cost-efficacy, and patient's health status. In addition, quality of life and sexual activity are relevant treatment aims, and they should be considered as outcome measures in trials comparing different treatment schedules. It is well known that different dose/fractionation regimens provide similar results in terms of local control, disease-free survival, and overall survival. However, other outcomes such as the impact of late toxicity on physical, mental, and social functioning should be considered. Some fractionation schedules could be inconvenient and detrimental to patient compliance, especially for elderly patients and for subjects living far from the radiotherapy center [25–30]. The 24 Gy in four weekly fraction schedule could represent a reasonable compromise between three and five fractions regimens with reduced inconvenience due to patient’s travels and potentially improved patient's satisfaction due to lower late toxicity rates.

This study has some limitations, including possible effects from occult bias due to the retrospective study design and underpowered analysis due to the low number of events. Another limitation is the differences in follow-up duration between the two groups, although this aspect might be negligible since vaginal toxicity usually occurs in the first years of follow-up. In fact, in our series, half of patients showed late toxicity events within one year after treatment.

## Conclusion

Four 6 Gy weekly fractions seem to be safer in terms of vaginal side effects and may be considered the better treatment between the two schedules. In fact, the two schedules compared in the present study showed significant differences in late vaginal toxicity rates, favoring 24 Gy/4 *versus* 21 Gy/3 fractions HDR-IRT. Larger studies with cost-effectiveness evaluations are needed to confirm the present results.
